# Five-year interim analysis of J-SKI: an observational study of TKI discontinuation in patients with CML in Japan

**DOI:** 10.1007/s12185-026-04184-4

**Published:** 2026-03-01

**Authors:** Naoto Takahashi, Shinya Kimura, Eri Matsuki, Takaaki Ono, Noriko Doki, Masaki Iino, Masashi Sawa, Yoshio Saburi, Kazunori Murai, Katsumichi Fujimaki, Shingo Kurahashi, Noriyoshi Iriyama, Takashi Onaka, Emiko Sakaida, Chikashi Yoshida, Keijiro Sato, Toshihiro Miyamoto, Tomoiku Takaku, Motoaki Shiratsuchi, Fumihiko Kimura, Seiichiro Katagiri, Matsuo Yamamoto, Akiko M. Saito, Hitoshi Kiyoi, Itaru Matsumura

**Affiliations:** 1 Department of Hematology, Akita University Hospital, 1-1-1 Hondo, Akita City, Akita 010-8543 Japan; 2 Saga University Hospital, Saga, Japan; 3 Keio University Hospital, Shinjuku-ku, Japan; 4 Hamamatsu University Hospital, Hamamatsu, Japan; 5 Tokyo Metropolitan Cancer and Infectious Diseases Center Komagome Hospital, Bunkyo-ku, Japan; 6 Yamanashi Prefectural Central Hospital, Kofu, Japan; 7 Anjo Kosei Hospital, Anjo, Japan; 8 Oita Prefectural Hospital, Oita, Japan; 9 Iwate Prefectural Central Hospital, Morioka, Japan; 10 Fujisawa City Hospital, Fujisawa, Japan; 11 Toyohashi Municipal Hospital, Toyohashi, Japan; 12 Nihon University Itabashi Hospital, Itabashi-ku, Japan; 13 NHO Saitama Hospital, Wako, Japan; 14 Kokura Memorial Hospital, Kitakyushu, Japan; 15 Japanese Red Cross Osaka Hospital, Osaka, Japan; 16 Chiba University Hospital, Chiba, Japan; 17 NHO Mito Medical Center, Ibaraki, Japan; 18 Japanese Red Cross Nagano Hospital, Nagano, Japan; 19 Kyushu University Hospital, Fukuoka, Japan; 20 Kanazawa University Hospital, Kanazawa, Japan; 21 Juntendo University Hospital, Bunkyo-ku, Japan; 22 Saitama Medical University Hospital, Iruma, Japan; 23 Iizuka Hospital, Iizuka, Japan; 24 National Defense Medical College Hospital, Tokorozawa, Japan; 25 Tokyo Medical University Hospital, Shinjyuku-ku, Japan; 26 NHO Nagoya Medical Center, Nagoya, Japan; 27 Nagoya University Hospital, Nagoya, Japan; 28 Kindai University Hospital, Osakasayama, Japan

**Keywords:** Chronic myeloid leukemia, Tyrosine kinase inhibitor, Treatment-free remission, Deep molecular response

## Abstract

Treatment-free remission (TFR) is an emerging goal for patients with chronic myeloid leukemia (CML) treated with tyrosine kinase inhibitors (TKI). However, long-term TFR durability in real-world settings remains understudied. The J-SKI study, a large observational study, was conducted to evaluate long-term TFR outcomes in Japanese patients with CML. This interim analysis included 795 eligible patients from the prospective (n = 283) and retrospective (n = 512) cohorts. With a median follow-up of 32 months (range 0.8–168) after TKI discontinuation, the 5-year TFR rate was 65.2% (95% confidence interval [CI]: 59.6–70.6%). Among patients who experienced molecular relapse, 99% (95% CI: 97–100%) regained a major molecular response after resuming TKI therapy, with no observed disease progression. Multivariate analysis identified the duration of deep molecular response (DMR) as the sole independent predictor of successful TFR, with each additional year of DMR reducing the relapse risk by 12.5% (HR: 0.875, *p* < 0.0001). A second TFR attempt was successful in 41% (95% CI: 22–59%) of the 32 patients. This study demonstrated that TKI discontinuation is a safe and feasible strategy in a real-world clinical setting. A sustained DMR is critical for TFR success, supporting its importance as a key therapeutic objective.

**Supplementary Information:**

The online version contains supplementary material available at 10.1007/s12185-026-04184-4.

## Introduction

Tyrosine Kinase Inhibitors (TKIs) have substantially improved the survival outcomes of patients with chronic myeloid leukemia in chronic phase (CML-CP). In clinical practice, TKI treatment is generally considered a lifelong commitment. Several clinical trials, including the largest prospective study, the European Stop Kinase Inhibitors (EURO-SKI) trial, have demonstrated that TKI therapy can be safely discontinued in approximately half of the patients who achieve and maintain a deep molecular response (DMR) [1–5]. In Japan, multiple prospective clinical studies have been conducted previously, demonstrating relatively short Treatment-free remission (TFR) periods of six months or one year [6–13]. Although a subset of Japanese patients treated with TKIs achieves sustained DMR and may be eligible for TFR, TFR long-term durability in real-world settings remains unclear. Furthermore, the clinical and biological factors predicting successful TFR are unclear, and the outcomes of a second TFR attempt remain understudied.

This large observational study was planned and conducted by the J-SKI Committee of the Japanese Society of Hematology (JSH) to identify the clinical conditions under which TFR is recommended and establish clinical practice guidelines for CML as evidence for the JSH guidelines.

## Patients and methods

### Study design and patients

The J-SKI’s primary objective, a multicenter observational study, was to determine the long-term TFR rate after TKI discontinuation in a large, real-world cohort of Japanese patients (UMIN 000037535). This study comprised two cohorts: a prospective TFR cohort of patients who discontinued TKI therapy in best clinical practice according to the 2018 JSH guidelines [14] and a retrospective TFR cohort of patients who had previously discontinued TKIs and were subsequently followed up prospectively. Patients with major *BCR::ABL1*-positive CML were enrolled from September 2019 to March 2025, with a data cut-off date of May 31, 2024. All the participants provided written informed consent. The key exclusion criteria included the inability to provide clinical information per the study schedule or participation in another clinical study in which data submission to this registry was not permitted.

### Study endpoints and assessments

The primary endpoint was the 5-year TFR rate after the discontinuation of TKI therapy. Secondary endpoints included the 10-year TFR rate, treatment-free survival (TFS), progression-free survival (PFS), and the cumulative incidence of regaining major molecular response (MMR; a *BCR::ABL1* international scale ≤ 0.1%) after treatment resumption. The exploratory endpoints were the identification of predictive factors for successful TFR and the success rate of a second TFR attempt.

To establish predictive factors that were associated with TFS, this study evaluated patients’ characteristics, including age, sex, additional chromosomal abnormalities (ACAs), Sokal score, EUTOS long-term survival (ELTS) score, prior interferon-α treatment, TKI as first line or just before TFR, switching of TKI, duration of TKI treatment, time to MMR, time to DMR, and duration of DMR. DMR was defined as MR4.5 (a *BCR::ABL1* international scale ≤ 0.0032%), presenting a 4.5-log reduction of *BCR::ABL1* mRNA in this study.

### Statistical analysis

Statistical analyses were conducted using the SPSS software version 28.0 Windows (IBM Corp., Armonk, NY, USA). The clinical characteristics of the patients are expressed as numbers or medians (minimum–maximum, or quartile 1–quartile 3). TFS was measured from the date of TKI discontinuation to the date of TKI re-administration or the date of the last molecular examination of patients with TFR. PFS was measured from the date of TKI discontinuation to the date of progression to an accelerated phase or blast crisis. TFS and PFS were estimated using the Kaplan–Meier method, and TFS was compared between the groups using the log-rank test. The rates of regained MMR after the resumption of treatment were calculated using the cumulative incidence method. To identify covariates that predicted a successful TFR, the proportion of patients with a TFR was calculated using Kaplan–Meier analysis, and a log-rank test was used to statistically compare the stratified groups (2 or more). Univariate and multivariate Cox regression analyses were performed. A stepwise multivariate approach was used to identify the most important prognostic factors with a two-sided variable retention criterion of *p* < 0.05. Variables that showed associations of marginal significance (two-sided *p* < 0.2) were used for the multivariate model building.

## Results

### Patients’ characteristics

Of the 844 registered patients, 795 were eligible for time-to-event analysis (prospective cohort, n = 283; retrospective cohort, n = 512). The baseline characteristics of the patients are summarized in Table 1. The median age was 51.0 years at diagnosis and 58.5 years at TKI discontinuation. Among the patients, 61.3% were male, and 98.1% were in the CP at diagnosis; 17.6% were classified as Sokal score high-risk, and 1.8% as ELTS high-risk at diagnosis. ACAs were observed in 7.3% of patients. The frequency of ACAs by karyotype is shown in Supplemental Table 1. Only 3.9% had a history of IFN-α administration before TKI therapy. Imatinib was selected as the first-line therapy for 46.0% of patients. However, 72.1% of the patients ultimately discontinued the second-generation TKI (2G-TKI) therapy, including those who switched from imatinib to 2G-TKI before TKI discontinuation. The median duration of TKI treatment was 85 months, and the median times to MMR and DMR were 11 and 24 months, respectively. The median durations of sustained MMR and DMR were 59 and 49 months, respectively. Because some cases used polymerase chain reaction (PCR) testing with MR4.0 sensitivity, 60 of 780 patients (7.5%) were recorded as having MR4.0 as DMR at TKI discontinuation, while 11 patients (1.4%) discontinued TKIs at the MMR level for various reasons.

**Table 1:** Patient backgrounds

Patient backgrounds	N = 795
Age at diagnosis [years], median (min–max)	51.0 (0–82)
Age at stopping TKI [years], median (min–max)	58.5 (5–94)
Sex, male, n (%)	487 (61.3%)
Chronic phase at diagnosis, n (%)	780 (98.1%)
Additional chromosomal abnormalities, n (%)	58 (7.3%)
Blast in peripheral blood at diagnosis [%], median (Q1–Q3)	0 (0–0.8)
*Sokal risk*	
Low, n (%)	226/443 (51.0%)
Intermediate, n (%)	139/443 (31.4%)
High, n (%)	78/443 (17.6%)
*ELTS*	
Low, n (%)	384/443 (86.7%)
Intermediate, n (%)	51/443 (11.5%)
High, n (%)	8/443 (1.8%)
Prior IFN-α treatment, n (%)	31 (3.9%)
TKI as the first line, IM/2G, n (%)	366 (46.0%)/429 (54.0%)
TKI switching, n (%)	288 (36.2%)
TKI just before TFR, IM/2G/3G, n (%)	209 (26.3%)/573 (72.1%)/13 (1.6%)
TKI duration [months], median (Q1–Q3)	85 (54–130)
Time to MMR [months], median (Q1–Q3)	11 (6–44)
Time to DMR [months], median (Q1–Q3)	24 (9–62)
MMR duration [months], median (Q1–Q3)	59 (37–90)
DMR duration [months], median (Q1–Q3)	49 (31–71)
MR4.5 as DMR at stopping TKI	724 (91.1%)
MR4.0 as DMR at stopping TKI	60 (7.5%)
MMR at stopping TKI	11 (1.4%)

N, number; ELTS, EUTOS long-term survival; IFN-α, interferon-alfa; 2G-TKI, second generation tyrosine kinase inhibitor; TFR, treatment-free remission; 3G, third generation; MMR, major molecular response; DMR, deep molecular response; Q1–Q3, quartile 1–quartile 3; MR4.5; *BCR::ABL1* international scale ≤ 0.0032%, MR4.0; *BCR::ABL1* international scale ≤ 0.01%

### TFR rate

**Fig. 1:**
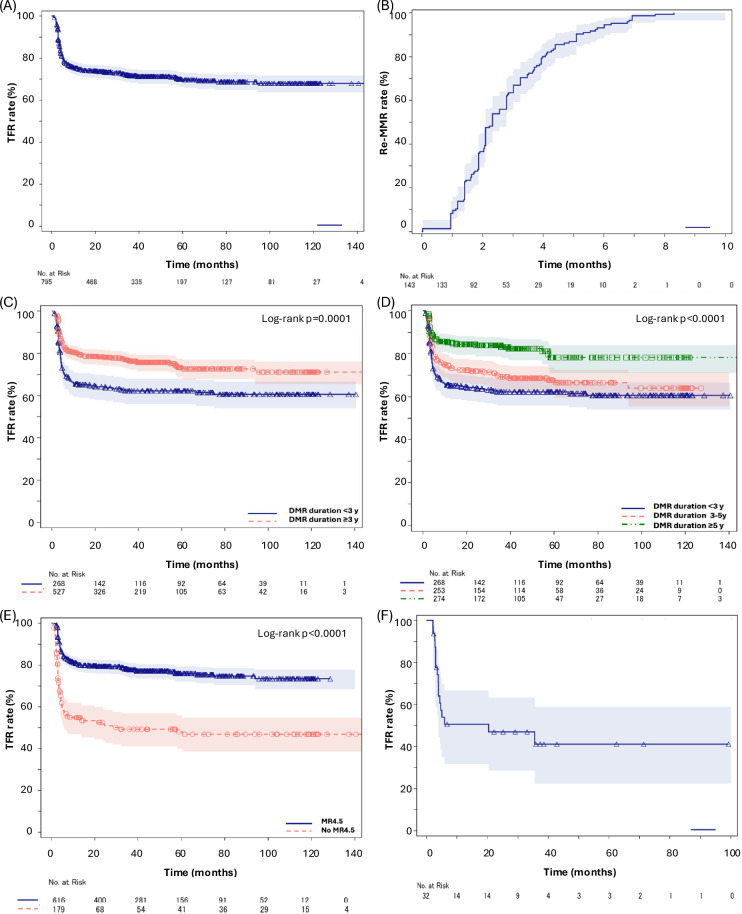
**A** Kaplan–Meier (KM) curve of treatment-free survival (TFS) after discontinuation of TKI in all patients, **B** Cumulative incidence of MMR regained after re-treatment by TKI (Re-MMR), **C** KM curve of TFS according to the duration of DMR (< 3 years vs. ≥ 3 years), **D** according to the duration of DMR (< 3 years vs. 3–5 years vs. ≥ 5 years), **E** according to the molecular status (MR4.5 vs. loss of MR4.5) at 3 months after TKI discontinuation, **F** KM curve of TFS after TKI discontinuation in patients of second attempt

With a median follow-up of 32 months (range, 0.8–168) from TKI discontinuation, the 5-year TFR rate and the 10-year TFR rate were 65.2% (95% confidence interval [CI]: 59.6–70.6%) and 60.6% (95% CI: 47.8–72.4%), respectively (Fig. 1A). At the cut-off date, patients experienced molecular relapse and resumed TKI therapy, and 99% (95% CI: 97–100%) subsequently regained MMR within 8 months (Fig. 1B). None of the patients progressed to an accelerated phase or blast crisis during the observation period.

### Predictive factors of successful TFR

Kaplan–Meier analysis revealed superior TFS in patients with longer duration of TKI (≥ 7 years) compared to those with shorter duration of TKI (< 7 years) (Supplemental Fig. 1D, *p* < 0.0001, by log-rank test). Patients with a longer DMR duration (≥ 3 years or ≥ 5 years) had superior TFS compared with those with a shorter DMR duration (< 3 years or 3–5 years) (Fig. 1C, *p* = 0.0001 and Fig. 1D, *p* < 0.0001). Even when focusing on the 321 cases who achieved TFR using only 2G-TKIs without switching TKIs, longer TKI duration (≥ 5 years) or DMR duration (≥ 3 years) was significantly associated with successful TFR (Supplemental Fig. 1I, *p* < 0.0001 and Supplemental Fig. 1J, *p* = 0.0032). There were no significant differences between the two groups of sex (Supplemental Fig. 1A), age (< 60 vs. ≥ 60), ACAs (Supplemental Fig. 1B), Sokal risk (Supplemental Fig. 1C), ELTS risk, TKI as the first line (Supplemental Fig. 1E), and TKI just before TFR (Supplemental Fig. 1F) using Kaplan–Meier analysis.

Conversely, sustained MR4.5 at 1, 3, or 6 months after TKI discontinuation was significantly associated with superior TFS compared to MR4.5 loss at 1, 3, or 6 months (Fig. 1E, *p* < 0.0001, Supplemental Fig. 1G, *p* < 0.0001, Supplemental Fig. 1H, *p* < 0.0001).

Molecular relapse risk and MMR loss after TKI discontinuation were analyzed using the Cox proportional hazards model based on clinical factors. Univariate analysis showed that molecular relapse risk decreased with each additional year of TKI treatment and the maintenance of MMR or DMR. Furthermore, a multivariate Cox regression analysis identified a longer duration of DMR as the sole independent predictor for successful TFR (HR: 0.875; 95% CI: 0.829–0.924; *p* < 0.0001), corresponding to a 12.5% relative reduction in molecular relapse risk for each additional year of DMR (Table 2).

**Table 2:** Cox regression analysis for predicting loss of MMR

Univariate		Expected	HR	95%CI	*P-*value
Sex	male versus female	0.17519	1.191	0.905–1.568	0.2114
Age at TFR [years]	< 60 versus ≥ 60	0.18432	1.202	0.923–1.566	0.1715
ACAs	yes versus no	0.34294	1.409	0.899–2.209	0.1348
Pre IFN-α	yes versus no	− 0.98042	0.375	0.140–1.008	0.0520
First line TKI	Imatinib versus 2G-TKI	− 0.19897	0.820	0.628–1.069	0.1429
TKI switching	no versus yes	0.09542	1.100	0.834–1.451	0.4988
TKI duration	1 year	− 0.08692	0.917	0.885–0.950	< 0.0001
MMR duration	1 year	− 0.09545	0.909	0.870–0.950	< 0.0001
DMR duration	1 year	− 0.13301	0.875	0.829–0.924	< 0.0001

Multivariate		Expected	HR	95%CI	*P*-value
Sex	male versus female	0.20720	1.230	0.934–1.621	0.1410
DMR duration	1 year	− 0.13383	0.875	0.829–0.924	< 0.0001

HR, hazard ratio; CI, confidence interval; TFR, treatment-free remission; ACAs, additional chromosomal abnormalities; IFN-α, interferon-alfa; TKI, tyrosine kinase inhibitor; 2G-TKI, second-generation tyrosine kinase inhibitor; MMR, major molecular response; DMR, deep molecular response

### Second attempt of TFR in J-SKI

A second TFR attempt was performed in 32 patients in whom the first attempt failed (Supplemental Table 2). The median time to loss of MMR after discontinuation of the first TKI was 112.5 days (range 32–1001 days). The median age was 60.0 years at the second attempt of TKI discontinuation. Among these 32 patients, 24 switched TKIs, mainly to 2G-TKI or third-generation TKI (3G-TKI), before the second TKI discontinuation attempt. The median duration of TKI treatment after readministration was 43 months, and the median duration of sustained DMR was 36 months. With a median follow-up of 5.5 months (range, 2–99 months) from the second TKI discontinuation attempt, the 6-month-TFR and subsequent TFR rates were 51% (95%CI: 32–67) and 41% (95%CI: 22–59), respectively (Fig. 1F). Although there was no statistically significant difference, a long duration of DMR (≥ 3 years) tended to be associated with better TFS by the Kaplan–Meier methods (Supplemental Fig. 1K, *p* = 0.0518). Additionally, MR4.5 at 3 months after 1st attempt of TKI discontinuation tended to be associated with better TFS using the Kaplan–Meier method (Supplemental Fig. 1L, *p* = 0.2004).

## Discussion

Here, we report the 5-year interim analysis results of the J-SKI study, which, to our knowledge, is the world’s largest observational study on TKI discontinuation. These results demonstrate that TKI treatment for CML-CP can be safely discontinued in routine clinical practice in Japan, with 60–70% of cases maintaining a molecular response for at least 5 years. This finding validates the results of the world’s largest EURO-SKI trial [3, 4] and confirms the outcomes of numerous Japanese TKI discontinuation studies that reported relatively short-term observation periods [6–13]. Furthermore, the Cox regression analysis in the J-SKI study identified DMR maintenance duration as an independent predictor of successful TFR. DMR maintenance duration has also been reported as a clinical factor for successful TFR in the EURO-SKI trial [3, 4]. Current guidelines such as ELN2025 and NCCN ver1. 2026 [15, 16] recommends a TKI treatment duration of at least five and three years, respectively, with a concurrent MR4 maintenance period of two years or more. However, it has been hypothesized that extending the duration of MR4 maintenance allows TKI to further eradicate minimal residual disease (MRD), even below the PCR detection limit, thereby leading to successful TFR in a greater number of patients. According to Michael Deininger’s hypothesis, based on the Stop Imatinib (STIM) trial [1], the deeper the level of the molecular response before TKI discontinuation, the longer the time to molecular relapse after discontinuation. Furthermore, given that relapses are rare after 7 months, he proposed the hypothesis that theoretically, once a certain level of molecular response—termed the “sustenance limit”—is exceeded, MRD is eradicated, and the relapse risk is virtually eliminated [17]. Consistent with previous reports [18, 19], the J-SKI study also showed that MR4.5 at 1, 3, and 6 months after TKI discontinuation was associated with the maintenance of long-term TFR. A deeper level of remission at the time of TKI discontinuation is considered a crucial clinical factor for TFR success.

The 5-year TFR rate in J-SKI was 65.2% (95% CI, 59.6–70.6), which is significantly higher than the 3-year TFR rate of 46% (95% CI, 42–49) in EURO-SKI [3, 4]. The most significant difference between these two studies lies in the definition of a DMR. In Japan, major *BCR::ABL1* international standard quantitative PCR kits provided by Otsuka Pharmaceutical (Tokyo, Japan) or Sysmex Corporation (Kobe, Japan) are available under insurance coverage, even in daily clinical practice, ensuring MR4.5 accuracy on the International Scale (IS-PCR). With a Limit of Detection (LOD) of 3–5 copies and a reference gene *ABL1* copy number of ≥ 150,000 copies, these are extremely high-sensitivity general-purpose tests [20, 21]. While J-SKI defines DMR as MR4.5, EURO-SKI defines DMR as MR4.0; therefore, the latter is thought to include cases that meet MR4.0 but have not achieved MR4.5. When comparing patient demographics between the EURO-SKI and J-SKI groups, there were no significant differences in the proportion of high-risk cases at diagnosis, duration of TKI treatment, or duration of DMR maintenance (Supplemental Table 3). However, DMR depth associated with its definition may be involved in TFR success. This result is considered to support the aforementioned "sustenance limit" hypothesis by Michael Deininger. Conversely, in the J-SKI analysis, the TKI type was not extracted as a factor for TFR success. Kaplan–Meier analysis showed that imatinib demonstrated a TFR rate comparable to that of 2G-TKIs, regardless of whether it was used as the first or last line of treatment. Although 2G-TKIs have a higher rate of early DMR achievement than imatinib and can ultimately increase the number of TFR candidates [22–24], evidence that 2G-TKIs enhance the TFR rate itself was not obtained from this study.

Prospective studies on rechallenge of TKI discontinuation are limited [25–28]. In this study, the clinical course of 32 cases of a second attempt was observed, and TFR was maintained in 40% of the cases. Although no statistically significant difference was observed, the duration of DMR maintenance before TKI discontinuation was considered an important factor in rechallenge, similar to the first TFR attempt. Furthermore, more than half of the 32 s-attempt cases attempted TKI discontinuation after switching from the initial TKI to another 2G or 3G-TKI. Future research outcomes regarding the rechallenge, such as the JALSG-Re STOP 219 study using 3G-TKIs (jRCTs041190117), are anticipated.

Notably, there are cases in which MMR is not lost over the long term, even if MR4.5 is lost by 6 months. This finding, demonstrated in the A-STIM trial [29], serves as the rationale for using the loss of MMR as a trigger for restarting treatment in subsequent STOP trials and in clinical practice. The existence of immunological effects that suppress the proliferation of MRD is speculated to be a force that maintains MMR [30]. Unfortunately, J-SKI lacks sufficient data on immunological factors, but an immunological sub-analysis of EURO-SKI has shown that NK cell activation is important for maintaining the TFR [31]. Additionally, previous TKI discontinuation studies have suggested the possibility of immunological effects, such as regulatory T cell suppression [32] and myeloid-derived suppressor cells (MDSCs) [33]. It is hoped that immunological analysis will predict patients who will succeed in TFR and that deeper DMR confirmed by high-sensitivity PCR will enable more cases to achieve TFR.

## Conclusion

The 5-year interim analysis of the J-SKI trial demonstrated that the TFR is a safe and feasible therapeutic goal in routine clinical practice for Japanese patients with CML. As the duration of DMR is a major independent predictor of successful TFR, future strategies should focus on the early achievement and maintenance of MR4.5. Therefore, 2G-TKIs or asciminib are recommended as first-line drugs, especially for patients with a high motivation for TFR. Furthermore, it is recommended to extend the MR4.5 maintenance period to ≥ 3 years. However, since some cases succeed in TFR even with the minimum condition of a 2-year MR4.5 maintenance period, the treatment duration should be determined through shared decision making (SDM), carefully weighing the disadvantages of continuing TKI against the increased success rate of TFR gained by continuation.

## Supplementary Information

Below is the link to the electronic supplementary material.

Supplementary file1 (PDF 173 KB)

Supplementary file2 (PDF 661 KB)

## Data Availability

Data will be made available on a reasonable e-request.
