# Supplementary material for: Five-year interim analysis of J-SKI: an observational study of TKI discontinuation in patients with CML in Japan
Source: Int J Hematol. 2026 Mar 1;124(1):96–103. doi: 10.1007/s12185-026-04184-4 (PMC13319150; doi:10.1007/s12185-026-04184-4)
Supplement: Supplementary file 1 — Supplementary file1 (PDF 173 KB) [file 12185_2026_4184_MOESM1_ESM.pdf]

Supplemental Table 1. Additional chromosomal abnormalities

**Karyotype of ACAs**	**N=58**	**%**
Second Ph chromosome	12	20.7
Trisomy 8	4	6.9
Isochromosome 17q	0	0.0
Trisomy 19	0	0.0
Complex chromosomal abnormalities	5	8.6
3q26.2 abnormalities	1	1.7
Others	44	75.9

Supplemental Table 2. Patient Background of second attempt for TFR

**Patient Background**	**N=32**
Age at diagnosis [years], median (min–max)	48.5 (24−77)
Age at stopping TKI [years], median (min–max)	56.5 (28−78)
Age at 2nd stop [years], median (min−max)	60.0 (32−82)
Sex, male, n (%)	20 (62.5%)
Sokal High, n (%) / ELTS High, n (%)	6 (32%) / 0 (0%)
Prior IFN-α treatment, n (%)	0 (0%)
TKI at 1st stop, IM vs. 2G-TKI, n (%)	4 (12.5%) vs. 28 (87.5%)
TKI duration before 1st stop [months], median (Q1–Q3)	54 (37−107)
DMR duration before 1st stop [months], median (Q1–Q3)	36 (17–53)
Time to loss of MMR during 1st attempt [days], median (Q1–Q3), range	112.5 (90.0–170.5), 32–1001
TKI at 2nd stop, IM vs. 2G-TKI vs 3G-TKI, n (%)	4 (12.5%) vs. 19 (59.4%) vs. 9 (28.1%)
TKI switching for 2nd attempt, n (%)	24 (75%)
TKI duration after Re-Ad [months], median (Q1−Q3)	43 (29−65)
MMR duration after Re-Ad [months], median (Q1−Q3)	38 (23−55)
DMR duration after Re-Ad [months], median (Q1−Q3)	36 (22−55)

N; number, TKI; tyrosine kinase inhibitor, ELTS; EUTOS long-term survival, IFN-α; interferon-alfa, 2G; second generation, 3G; third generation, DMR; deep molecular response, Q1−Q3; quartile 1-quartile 3, Re-Ad; re-administration, MMR; major molecular response, DMR; deep molecular response, Q1-Q3; quartile 1−quartile 3.

Supplemental Table 3. Comparison of Patient Backgrounds in J-SKI and EURO-SKI

Patient backgrounds	J-SKI (N=795)	EURO-SKI (N=728)
Age at diagnosis [years], median (min–max)	51.0 (0–82)	52 (11–85)
Age at stopping TKI [years], median (min–max)	58.5 (5–94)	60 (19–89)
Sex, male, n (%)	487 (61.3%)	386 (53%)
Chronic phase at diagnosis, n (%)	780 (98.1%)	NA
Additional chromosomal abnormalities, n (%)	58 (7.3%)	NA
Blast in PB [%], median (min–max)	0 (0–72.5)	0 (0–18)
Sokal risk		
Low, n (%)	226 /443 (51.0%)	253/562 (45.0%)
Intermediate, n (%)	139 /443 (31.4%)	197/562 (35.1%)
High, n (%)	78 /443 (17.6%)	112/562 (19.9%)
ELTS		
Low, n (%)	384/443 (86.7%)	399/562(71.0%)
Intermediate, n (%)	51/443 (11.5%)	124/562 (22.1%)
High, n (%)	8 /443 (1.8%)	39/562 (6.9%)
Prior IFN-α treatment, n (%)	31 (3.9%)	126 (17.4%)
TKI as the first line, IM, n (%)	366 (46.0%)	682 (93.7%)
TKI switching, n (%)	288 (36.2%)	117 (16.1%)
TKI just before TFR, IM, n (%)	209 (26.3%)	579 (79.5%)
TKI duration [years] median, (min–max)	7.1 years (0.3-21.5)	7.5 (3.0-14.1)
DMR duration [years] median, (min–max)	4.1 years (0-17.5)	4.7 (1.0-13.3)
MR4.5 in DMR at stopping TKI	724 (91.1%)	531 (72.9%)
MR4.0 in DMR at stopping TKI	60 (7.5%)	197 (27.1%) *
MR3.0 at stopping TKI	11 (1.4%)	0

TKI; tyrosine kinase inhibitor, PB; peripheral blood, IFN-α; interferon-α, IM; imatinib, TFR; treatment free remission, DMR; deep molecular response.

*Everyone was in MR4.0, at least. There had not been enough reference genes to confirm MR4.5 in the 146 of 197 patients.
