# Supplementary material for: Five-year interim analysis of J-SKI: an observational study of TKI discontinuation in patients with CML in Japan
Source: Int J Hematol. 2026 Mar 1;124(1):96–103. doi: 10.1007/s12185-026-04184-4 (PMC13319150; doi:10.1007/s12185-026-04184-4)
Supplement: Supplementary file 2 — Supplementary file2 (PDF 661 KB) [file 12185_2026_4184_MOESM2_ESM.pdf]

Supplemental Fig.

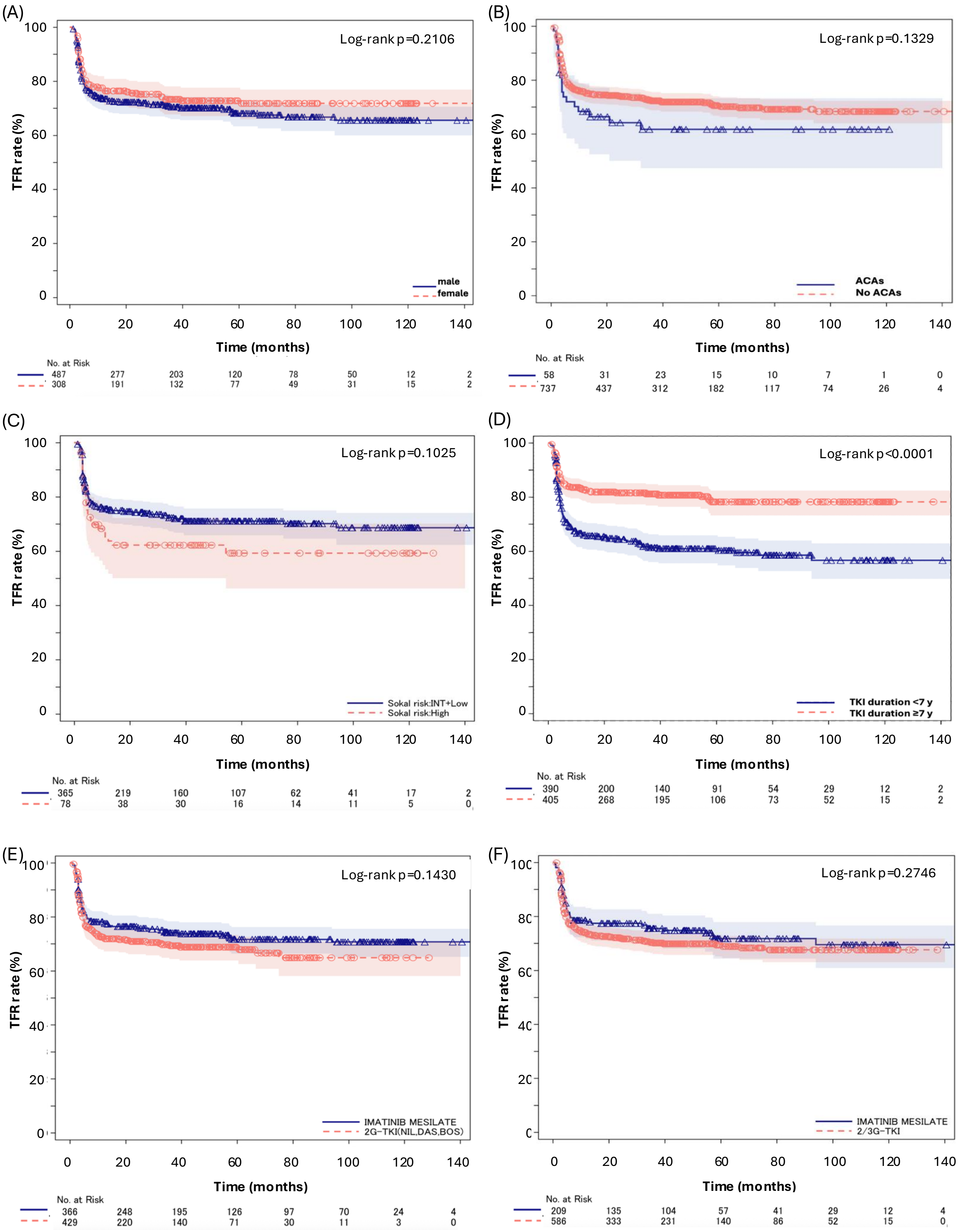

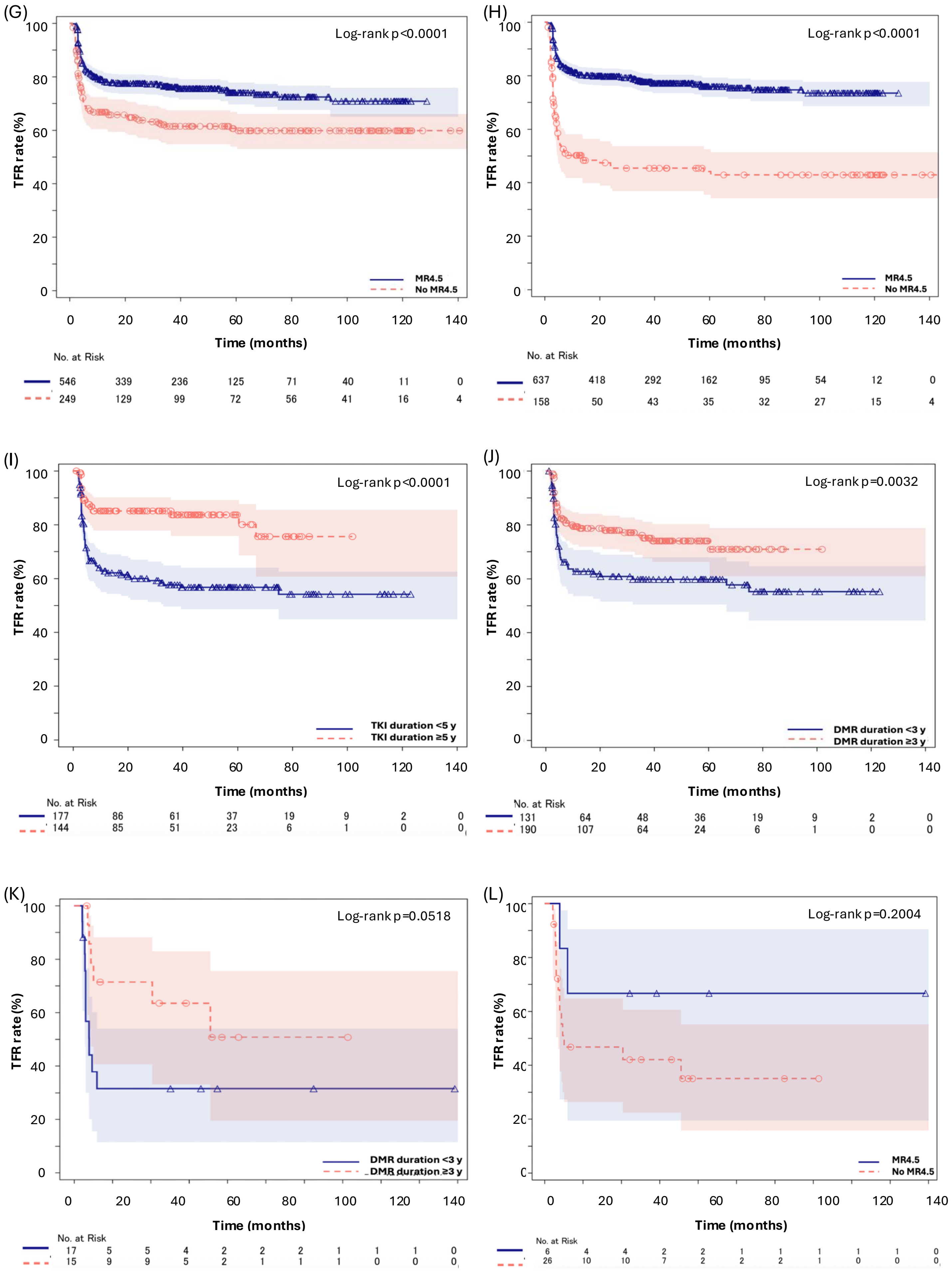

(G)
Log-rank p<0.0001
TFR rate (%)
Time (months)
MR4.5
No MR4.5
No. at Risk
546 339 236 125 71 40 11 0
249 129 99 72 56 41 16 4
(H)
Log-rank p<0.0001
TFR rate (%)
Time (months)
MR4.5
No MR4.5
No. at Risk
637 418 292 162 95 54 12 0
158 50 43 35 32 27 15 4
(I)
Log-rank p<0.0001
TFR rate (%)
Time (months)
TKI duration <5 y
TKI duration ≥5 y
No. at Risk
177 86 61 37 19 9 2 0
144 85 51 23 6 1 0 0
(J)
Log-rank p=0.0032
TFR rate (%)
Time (months)
DMR duration <3 y
DMR duration ≥3 y
No. at Risk
131 64 48 36 19 9 2 0
190 107 64 24 6 1 0 0
(K)
Log-rank p=0.0518
TFR rate (%)
Time (months)
DMR duration <3 y
DMR duration ≥3 y
No. at Risk
17 5 5 4 2 2 2 1 1 1 0
15 9 9 5 2 1 1 1 0 0 0
(L)
Log-rank p=0.2004
TFR rate (%)
Time (months)
MR4.5
No MR4.5
No. at Risk
6 4 4 2 2 1 1 1 1 1 0
26 10 10 7 2 2 2 1 0 0 0

(A) Kaplan–Meier (KM) curve of treatment-free survival (TFS) after discontinuation of TKI in all patients according to sex, (B) according to additional chromosomal abnormalities, (C) according to Sokal risk, (D) according to the duration of TKI (<7 years vs. ≥ 7 years), (E) according to TKI as the first line (IM vs. 2G-TKI), and (F) according to the final TKI just before TFR. (G) according to the molecular status (MR4.5 vs. loss of MR4.5) at 1 months after TKI discontinuation, (H) according to the molecular status (MR4.5 vs. loss of MR4.5) at 6 months after TKI discontinuation, (I) according to duration of TKI (<5 years vs. ≥ 5 years) among patients treated with 2G-TKI as the first line treatment without switching TKI, (J) according to the duration of DMR (<3 years vs. ≥ 3 years) among patients treated with 2G-TKI as the first line treatment without switching TKI, (K) KM curve of TFS after discontinuation of TKI in patients of second attempt according to the duration of DMR (<3 years vs. ≥ 3 years) after re-treatment, (L) according to the molecular status at 3 months after 1st TFR attempt (MR4.5 vs. loss of MR4.5).
